# Effects of Thermal and Antibiotic Treatments on the Viral Accumulation of FcMV1 in *Fusarium circinatum* Isolates

**DOI:** 10.3390/jof11040267

**Published:** 2025-03-31

**Authors:** Huma Amin, Cristina Zamora-Ballesteros, Julio Javier Diez-Casero

**Affiliations:** 1 Department of Plant Production and Forest Resources, Sustainable Forest Management Research Institute (iuFOR), Higher Technical School of Agricultural Engineering (ETSIIAA), University of Valladolid, 34004 Palencia, Spain; 2 Faculty of Environment and Natural Resources, Albert-Ludwigs-Universität Freiburg, Bertoldstraße 17, 79098 Freiburg im Breisgau, Germany

**Keywords:** mycoviruses, biological control, hypovirulence, forest pathology, pine pitch canker, mitoviruses

## Abstract

Mycoviruses are viruses that infect fungi, including plant pathogens. The infection of these mycoviruses is sometimes associated with impaired phenotypes of their fungal hosts, a phenomenon known as hypovirulence. Thus, using mycoviruses as biological control agents has emerged as a promising tool to combat forest diseases. The invasive ascomycete fungus *Fusarium circinatum*, which causes pine pitch canker (PPC) disease in *Pinus* tree species and other coniferous trees, is infected by the mycovirus *Fusarium circinatum* mitovirus 1 (FcMV1), FcMV2-1, and FcMV2-2. However, its impact on pathogen fitness remains unclear. The most accurate method used to identify the effect of a mycovirus on its host is the generation of isogenic lines with and without the mycovirus. The present study aimed to cure *F. circinatum* isolates infected by FcMV1 using different approaches. For this purpose, three replicates of each isolate were exposed to thermal treatment (38 °C) and antibiotic treatment (ribavirin, cycloheximide, kanamycin, and rifampicin mixed with cAMP)(cyclic adenosine monophosphate) for five successive passages. The viral titer of FcMV1 was then assessed using qPCR (quantitative polymerase chain reaction) after the first week and after the fifth week of the treatment. The results revealed differences in treatment efficacy among *F. circinatum* isolates, with some showing very low virus titers at the end of the experiment. Both thermal and antibiotic treatment effectively reduced the viral load in all isolates. In addition, the antibiotic cycloheximide and rifampicin +cAMP reduced the viral titer more than ribavirin and kanamycin. The isolate Fc179 was found to be more prone to antibiotic treatment than the other two isolates (001 and Va221). This study demonstrated the possibility of using some isolates of *F. circinatum* for fine-tuning cures for mitovirus, in order to create virus-free strains for biological control in the future.

## 1. Introduction

The estimated number of fungal species on Earth is approximately 1.5 million, with 30–80% of these species potentially hosting viral infections [1]. Viruses that infect fungi, known as mycoviruses or fungal viruses, are ubiquitous and found across all major fungal groups [1,2,3,4]. Since the first description of mycoviruses, over 250 mycovirus genomes have been fully or partially sequenced [5]. The International Committee on Taxonomy of Viruses (ICTV) has categorized mycoviruses into twelve single-stranded RNA families, nine double-stranded RNA families, two reverse transcription virus families, and one ssDNA family [6]. Mycoviruses are notable for their distinctive characteristics, including unique genomic and virion structures, as well as antiviral defense mechanisms which set them apart from prevalent plant and animal viruses [7,8]. Fungi are susceptible to viral infections not only from closely related viruses but also from distantly related viral families [9].

Mycoviruses, like plant and animal viruses, rely on host cells for replication. However, unlike many other eukaryotic viruses, they are generally not transmitted through extracellular routes [10]. Instead, mycoviruses are transmitted intracellularly through cells or hyphal fusion (horizontal transmission) and sporulation (vertical transmission) [2,5]. The effects of most mycoviruses on the biology of their fungal hosts in the natural environment are still unknown. However, some mycoviruses cause either negative or positive effects, such as altering spore production (sexual/asexual), pigmentation and growth or, in the case of fungal pathogens, affecting virulence (hypo- and hyper-virulence) [11,12,13]. For instance, the co-infection of *Monilinia fructicola* with three distinct mycoviruses led to enhanced growth in vitro compared to the virus-cured isogenic lines of *M. fructicola*. However, no significant differences were observed on prunes infected with either *M. fructicola* or isogenic fungal lines, suggesting that these mycoviruses did not affect the pathogen’s virulence on the host [14].

Virocontrol, a biological control strategy that involves using mycovirus-induced hypovirulence to attenuate the virulence of phytopathogenic fungi, has gained significant attention from researchers over recent decades [15,16,17,18]. A notable illustration of hypovirulence is observed with *Cryphonectria* hypovirus 1 and 2 (CHV1, CHV2) from the *Hypoviridae* family, which induces hypovirulence in *Cryphonectria parasitica,* the causal agent of chestnut blight disease [19,20]. In particular, CHV1 was found to cause phenotypic changes in fungal hosts, including growth reduction and altered pigmentation [16]. In addition to CHV1 and CHV2, several other mycoviruses causing hypovirulence have been identified in pathogenic fungi such as *Fusarium graminearum*, *Botrytis cinerea*, *Sclerotinia sclerotium*, and *Rosellinia necatrix* [21,22,23,24]. The successful application of hypovirulence-induced mycovirus to control chestnut blight in Europe has prompted further research into the mycovirome of pathogenic fungi that infects tree species [25]. However, the effects of most mycoviruses on fungi hosts remain unclear; however, these viruses can be characterized as difficult to cure [5,11,26,27].

*Fusarium circinatum* is an invasive fungal pathogen responsible for causing pine pitch canker (PPC) disease, which poses a significant threat to nurseries, plantations, and natural forests of conifers [28,29]. Various management strategies, such as the removal or cutting of infected trees, quarantine measures, or sanitation practices, have failed to effectively control PPC [30,31,32]. This, together with the detection of three mycoviruses (FcMV1, FcMV2-1, and FcMV2-2) infecting *F. circinatum*, prompted the investigation of the possibility of virocontrol as a potential strategy against PPC. These mycoviruses belong to the genus Mitovirus and family Narnaviridae; which are abundant in filamentous fungi and predominantly localized in the mitochondria of their host, utilizing mitochondrial translation machinery [8,33]. Additionally, the *F. circinatum* mitoviruses FcMV1, FcMV2-1, and FcMV2-2 possess positive-sense single-stranded RNA ((+) ssRNA) genomes ranging from 2 to 3 kb in length, which have a single open reading frame (ORF) encoding an RNA-dependent RNA polymerase (RdRp) [29].

Preliminary studies on *F. circinatum* mitoviruses have not exposed any evident hypovirulence-inducing characteristics [34,35]. Further studies are needed to elucidate the possible role of these mitoviruses on the PPC pathogen. The most accurate method used to identify the impact of mycoviruses on their hosts is to generate isogenic lines of the fungal host with and without the infection of these viruses [36]. Previous virus-curing approaches have usually involved growing cultures using single spore isolation, hyphal tips, or protoplast isolation [37,38], the treatment of fungal cultures at higher or lower temperatures [14], or the use of various antibiotics to inhibit virus replication [39]. The antibiotic cycloheximide is widely used to eliminate RNA mycoviruses [40], but this approach still needs to be investigated, as curing is not always successful. In one study, *Aspergillus niger* infected with several virus-like particles treated with cycloheximide failed to eliminate any of the viruses [41]. In contrast, the same antibiotic (cycloheximide) was successful in eliminating mycoviruses from *Aspergillus fumigatus* partitivirus-1 [42], *Lentinula erodes* [43], and partially from *Pseudogymnoascus destructans* [44]. In this study, we determined the effects of thermal and antibiotic treatments on the virus titer accumulation of *F. circinatum* mitovirus FcMV1 to create isogenic or virus-free isolates for possible use as biocontrol agents.

## 2. Materials and Methods

### 2.1. Fungal Isolates and Mycoviruses

Three different Spanish isolates of *F. circinatum* (001, VA221, and Fc179) naturally infected with mitovirus FcMV1 were used in this study [33,45,46]. The isolate FcCa6 was used as a positive control because it is also infected with FcMV1 [47]. All the isolates were cultured on potato dextrose agar medium (PDA; Difco Laboratories, Detroit, MI, USA) at 28 °C for seven days. Before starting the experiment, the presence of mycoviruses was confirmed using qPCR with virus-specific primers (Table S1). Following this initial incubation, each colony was transferred onto a PDA medium with a cellophane disc for further analysis.

### 2.2. Mycovirus-Curing Treatments

Different approaches were tested to eliminate FcMV1 from each fungal isolate, including exposure to high temperatures and treatment with various antibiotics. Each treatment was applied to three biological replicates of each fungal isolate.

#### 2.2.1. Thermal Treatment

A preliminary test was conducted on each fungal isolate to determine its sensitivity to various temperatures (28 °C to 38 °C). The fungus was able to grow until 38 °C but could not survive beyond this threshold. Consequently, 38 °C was established as the maximum temperature for thermal treatments. To initiate the experiment, a 4 mm mycelium plug of the actively growing fungal isolate was subcultured onto fresh PDA plates and incubated at 28 °C for 4 days for hyphal growth. Then, the plates were re-exposed to thermal treatment at 38 °C for another seven days to evaluate the persistence of mycovirus. The colonies of thermally treated plates were subcultured onto fresh PDA plates and simultaneously onto cellophane-covered PDA medium to further check for the presence of the mycovirus. In addition, the colonies from the thermally treated plates were also subcultured onto fresh PDA plates and kept at 28 °C for 4 days for hyphal growth. After 4 days, the plates were incubated again at 38 °C for seven days to determine the presence of mycovirus. The plates were exposed for intermittent exposure at 38 °C to reduce virus accumulation. Thermal treatment was repeated for five consecutive weeks for all three biological replicates of each isolate. The virus titer was analyzed during the first and fifth weeks of treatment.

#### 2.2.2. Antibiotic Treatment

The fungus sensitivity to each antibiotic was tested prior to the experiment. The antibiotics used for the subsequent experiment were ribavirin (24 mg/L), cycloheximide (20 mg/L), kanamycin (50 mg/L), and rifampicin 25 mg/L mixed with cyclic adenosine monophosphate (cAMP) (0.33 mg/L) [36,48]. A 4 mm mycelium plug was cultured on PDA plates supplemented with the respective antibiotics. Colonies were subcultured at seven-day intervals on fresh PDA supplemented with antibiotic plates and on cellophane-covered PDA plates for further analysis. The presence of mycelium was tested in the first and fifth weeks of treatments. Additionally, fungal cultures were transferred to antibiotic-free PDA ‘recovery plates’, incubated in the dark at 25 °C for seven days, and then tested again for the presence of the mitovirus.

#### 2.2.3. Effect of Mycoviruses on Fungal Growth

All three isolates after thermal and antibiotics treatments were analyzed to ensure the fungal growth. A 4 mm mycelium plug from treated fungal hyphae was cultured onto fresh PDA plates and incubated at room temperature (28 °C) for seven days. Similarly, wild-type strains of all three isolates were inoculated onto PDA plates and incubated under the same conditions. After seven days, the radial fungal growth was observed visually for both wild-type and treated isolates. The treated isolates exhibited reduced growth compared to their wild-type counterparts.

### 2.3. RNA Extraction and cDNA Synthesis

Approximately 100 mg of mycelium grounded with liquid nitrogen was used for RNA extraction. Total RNA from all isolates was extracted using the Spectrum™ Plant Total RNA Kit (Sigma Aldrich, St. Louis, MO, USA) following the manufacturer’s protocols. The genomic DNA was removed from the samples using on-column DNase Digestion (DNASE10-1 SET, Sigma-Aldrich, St. Louis, MO, USA). The quantity and integrity of RNA were measured using the Invitrogen Qubit 4 Fluor meters (Qubit™, Thermo Fisher Scientific, Waltham, MA, USA) and gel electrophoresis (1% TAE), respectively. Reverse transcription (cDNA synthesis) was synthesized in 20 µL reactions using a 1 µg RNA template, 1 µL of 5 mM dNTPs, and 1 µL of random hexamers (10X) (Thermo Scientific™ Random Hexamer Primer, Waltham, MA, USA). To synthesize first-strand cDNA, the reactions were incubated at 65 °C for 5 min in a thermocycler’s first step. For the second step, reactions were incubated at 25 °C for 10 min for primer annealing, then at 65 °C for 60 min for strand extension, followed by incubation at 85 °C for 5 min to denature the enzyme.

### 2.4. Mycovirus Detection Through qPCR

For the detection and quantification of the mitovirus FcMV1, quantitative PCR (qPCR) was used. Virus-specific primers (Table S1) were used to amplify a region of open reading frame (ORF) coding for RNA-dependent RNA polymerase (RdRp) of the mitovirus [45]. Primers targeting *β*-*tubulin* of the pathogen were used as housekeeping genes. For qPCR setup, each reaction was prepared by mixing 1 uL cDNA, 0.8 μL (10 μM) of each primer, and 10 μL of PowerUp™ SYBR™ Green Master Mix (Thermo Fisher Scientific), rounding up to 20 μL with nuclease-free water. Amplifications were carried out using the QuantStudio 6 Flex Real-time PCR System (Applied Biosystems, Thermo Fisher Scientific) using a protocol consisting of an initial activation step at 95 °C for 3 min, and 40 cycles of 94 °C for 15 s, 60 °C for 20 s, and 72 °C for 30 s. All qPCR experiments were conducted in duplicate. The Cycle threshold (Ct) values represent the number of amplification cycles required for the fluorescence signal to exceed the baseline threshold. Ct values are inversely proportional to the viral load, meaning that higher Ct values indicate lower viral concentrations [49]. Ct values were determined with the QuantStudio Real-Time PCR v1.7.1 software. The relative quantification of viral RNA was assessed using the 2^−ΔΔCt^ (RQ) method by normalizing virus-specific ORF amplification levels to the housekeeping gene at the first and fifth weeks of treatment. ΔΔCt values were calculated by comparing the ΔCt of treated samples to untreated controls. The RQ value represented the fold change in viral RNA load in treated samples relative to the control [49]. The average Ct values were used across three biological replicates for each isolate.

### 2.5. Statistical Analysis

The variables considered in this study were treatment types (thermal treatment, antibiotic treatment, and positive control) and assessment time points (Week 1 and Week 5 post-treatment). The difference in Cts before and after treatment at Week 5 was tested using a paired *t*-test (significant, *p*-value < 0.05). A paired *t*-test (significant, *p*-value < 0.05) was also used to measure the difference in viral accumulation between treated and control samples. A single-factor ANOVA test was performed to analyze the effects of treatments across the isolates at Week 5.

## 3. Results

### 3.1. Confirmation of FcMV1 Infection Before Treatments

The presence of mycoviruses in each of the three isolates was analyzed using qPCR, by analyzing the amplification levels and melting temperature. Ct values from the amplification curves of all isolates are shown in Table 1. The specificity analysis was conducted by comparing the melting temperature of the different isolates to that of positive sample (FcCa6). All isolates used in the experiment showed the same melting temperature, 77.24 °C (±0.1), as the positive isolate (Figure S1). Additionally, the NTC (no template control) showed neither an amplification curve nor a melting curve, confirming the presence of the mitovirus FcMV1 in the three *F. circinatum* isolates before the start of virus-curing treatments.

### 3.2. Stability of Mitovirus FcMV1 After Thermal Treatment

**Figure 1:**
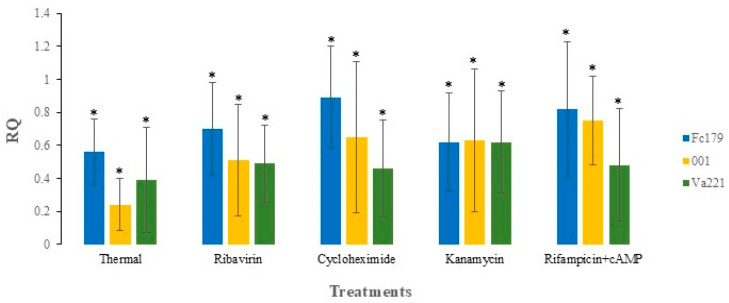
The reductions in viral titer in each isolate (Fc179, 001, and Va221) after thermal and antibiotic treatments. The value represents the fold change, which shows the % of viral accumulation in all isolates after treatments at W5. The level of statistical significance was determined using a paired *t*-test, *: *p* < 0.05.

**Figure 2:**
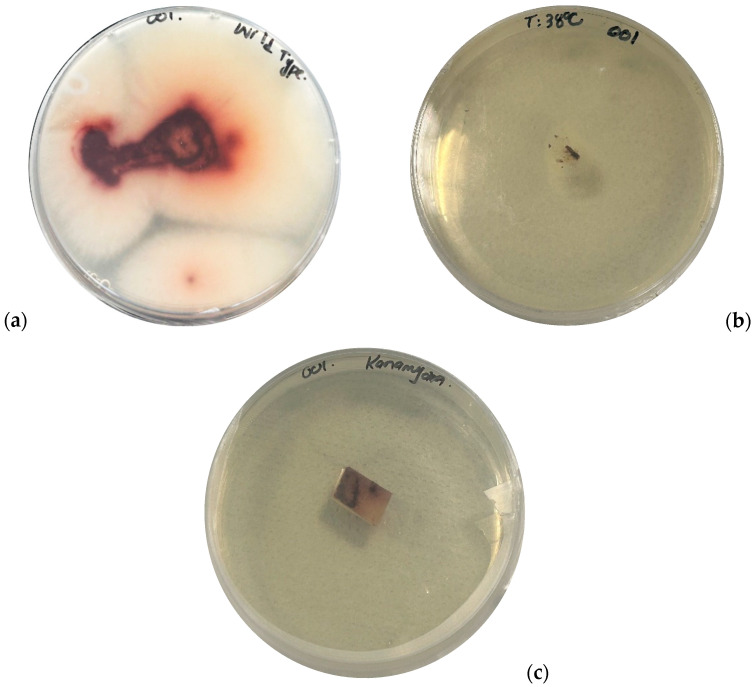
Representation of reduction in radial fungal growth in treated isolates. (**a**) represent the wild-type isolate 001. (**b**) represents isolate 001 treated thermally at 38 °C, and (**c**) represents isolate 001 treated with the antibiotic kanamycin.

The presence or accumulation of the virus in the treated isolates was tested as previously described. Ct values of isolates at week one (W1) and week five (W5) (Table S2) were compared and assessed with the Ct values recorded for the confirmation of FcMV1 presence before treatment (Table 1) to assess changes in viral load. Thermal treatment effectively reduced the viral accumulation in all isolates. In isolate Fc179, the mean Ct value of all the replicates increased from 17.7 (pre-treatment) to 22.7 after the first week (W1), indicating a reduction in viral load, as higher Ct values imply a lower DNA load. By the fifth week (W5), viral accumulation in the isolate Fc179 was further reduced, with a significant increase in Ct to 29.6 (*p* < 0.05). Therefore, the Ct value for virus-specific primers at W5 was normalized by the housekeeping gene using the fold change method (Section 2.4). The fold change (RQ) value calculated for isolate Fc179 was 0.56 (*p* < 0.05), indicating a 56% reduction in viral accumulation compared to the control. Similarly, the Ct value of isolate 001 increased to 23.5 after W1 treatment, indicating a reduction in viral load. By W5, the Ct value further increased to 29.6 (*p* < 0.05), confirming a significant decrease in viral accumulation. The RQ value calculated was 0.24 (*p* < 0.05) for isolate 001, implying a 24% reduction in viral accumulation compared to the control. In addition, isolate Va221 exhibited a higher Ct value (Ct = 23.1) after W1 of the treatment, leading to the significant reduction in viral load at W5 with the Ct value (Ct = 29.2) (*p* < 0.05). Consequently, the calculated RQ value of isolate Va221 was 0.39 (*p* < 0.05), indicating a 39% reduction in viral accumulation of the treated sample compared to the control. After five consecutive rounds of the treatment, virus-specific assays confirmed reductions in viral titer across all isolates (Figure 1). Furthermore, isolates subjected to the thermal treatment exhibited a reduction in radial growth compared to wild-type isolates (Figure 2). A single-factor ANOVA was used to determine the level of statistical significance among the treated isolates at W5 (Table 2). Although no statistically significant differences were found between the treatments of isolates (*p* = 0.12), numerically, the thermal treatment was found to be more effective on isolate Fc179(56%), followed by isolate Va221 (39%) and isolate 001 (24%). 

### 3.3. Stability of Mitovirus FcMV1 After Antibiotic Treatment

The presence or accumulation of the mitovirus FcMV1 was assessed after the exposure of isolates to four different types of antibiotics. The average Cts of all three isolates at W1 and W5 (Table S2) were compared to the Cts of the isolate calculated before the treatments (Table 1). In isolate Fc179, treatment with ribavirin resulted in a reduction in viral load at W1 (22.5), with a significant reduction at W5 (27.9; *p* < 0.05), in comparison with the Ct of isolate Fc179 before treatments (Ct = 17.7). The fold change analysis (RQ) confirmed a 70% reduction in viral load (*p* < 0.05) at W5 compared to the control. Similarly, in isolate 001, ribavirin treatment resulted in an increase in Ct from 18.2 (before treatment) to 21.7 at W1, reflecting a decrease in viral load. However, at W5, isolate 001 showed a significant reduction in viral load, with its Ct value increasing to 29.0 (*p* < 0.05). This corresponded to a fold change (RQ) value of 0.51, which accounts for a 51% (*p* < 0.05) reduction in viral accumulation in treated vs. control isolates. In addition, Ct values increased from 19.6 (before treatment) to 23.1 at W1 and further to 23.7 at W5 (*p* < 0.05).

The treatment with antibiotic cycloheximide showed a reduction in viral load in isolate Fc179, with Ct values increasing from 17.7 (before treatment) to 26.0 at W1 and 27.8 at W5 (*p* < 0.05). This indicated a significant decrease in viral accumulation. In addition, the fold change analysis (RQ = 0.89) confirmed an 89% (*p* < 0.05) reduction in viral accumulation compared to the control.

Isolate 001, when treated with cycloheximide, represented a significant decrease in viral load at both W1 and W5 (Ct W1 = 23.4 and Ct W5 = 28.0; *p* < 0.05), compared to the same isolate before treatment (Ct = 18.2). This decrease was further confirmed by a fold change value of 0.65, indicating a 65% (*p* < 0.05) reduction in viral accumulation. Similarly, for isolate Va221, the Ct values increased from 17.7 (before treatments) to 22.0 at W1 and 28.9 at W5 (*p* < 0.05), demonstrating a decrease in viral load after cycloheximide treatment. The fold change value calculated for isolate Va221 at W5 was 0.46, describing a 46% (*p* < 0.05) reduction in viral accumulation after treatment.

Kanamycin has been minimally explored for virus-curing studies. Still, we found that isolate Fc179, when treated with kanamycin, showed a reduction in viral load at W1 (Ct = 23.3) and W5 (27.3; *p* < 0.05), respectively, compared to the Ct before treatment (17.7). This decrease corresponded to a fold change value for isolate Fc179 of 0.62, indicating a 62% (*p* < 0.05) reduction in virus accumulation. Likewise, isolate 001, when treated with kanamycin, showed a similar decrease in viral load at W1 and W5 (Ct = 23.2 and Ct = 27.5; *p* < 0.05). However, the Ct of isolate 001 was (18.2) before treatment. Furthermore, the fold change value for isolate 001 was 0.63, depicting a 63% (*p* < 0.05) reduction in viral accumulation. Likewise, the isolate Va221, when treated with kanamycin, demonstrated a viral load reduction at W1 and W5 (Ct = 22.2 and Ct = 27.4; *p* < 0.05) compared to isolate Va221 before treatment (Ct = 19.6), corresponding to a fold change value of 0.62, which demonstrated a 62% (*p* < 0.05) reduction in viral accumulation.

The isolates treated with Rifampicin +cAMP exhibited a reduction in viral accumulation. The isolate Fc179, when treated with Rifampicin +cAMP, demonstrated a reduction in viral load at W1 (Ct = 20.8), with a significant reduction at W5 (Ct = 27.4; *p* < 0.05), compared to the Ct of the isolate before treatment (Ct = 17.7). This decline corresponded to a fold change value of 0.82, depicting an 82% (*p* < 0.05) reduction in viral accumulation compared to the control. Similarly, the viral load for isolate 001, when treated with rifampicin +cAMP, was reduced at W1 and W5 (Ct = 21.1 and 27.1; *p* < 0.05), compared to the Ct of the isolate before treatment (Ct = 18.2). In addition, the RQ value of 001 (0.75) showed a 75% (*p* < 0.05) reduction in viral accumulation. Likewise, the treatment of isolate Va221 with Rifampicin +cAMP reduced its viral load at both W1 and W5 (Ct = 22.5 and Ct = 28.4; *p* < 0.05), compared to the Ct of Va221 before treatment (Ct = 19.6). The corresponding fold change value calculated for Va221 was 0.48, representing a 48% (*p* < 0.05) reduction in viral accumulation. After five rounds of antibiotic treatments, the virus-specific analysis revealed that all treatments effectively reduced the virus titer (Figure 1).

In addition, isolates treated with antibiotics showed inhibited radial fungal growth compared to wild-type isolates (Figure 2). However, the interactions between the treated isolates with all antibiotics at W5 were determined using a two-factor ANOVA with replication; statistically, there was no difference between the treatments. However, numerically, we found that isolate Fc179 was more affected by antibiotic treatment, followed by isolate 001 and Va221.

## 4. Discussion

The detection of hypovirulence-induced mycovirus has advanced significantly due to the extensive use and development of RNA-sequencing technologies [26]. However, studying the effects on their hosts requires mycovirus manipulation, which is often complex. This study represented an attempt to eliminate the mitovirus FcMV1 from the pathogenic fungus *F. circinatum* using thermal treatment (38 °C) and exposure to various antibiotics. However, the effectiveness of these approaches varied, depending on the fungal isolate. Mycovirus infections are mostly persistent and challenging to eliminate from their hosts [50,51]. Various attempts to eliminate or cure viruses from fungi using several approaches, including hyphal tip transfer, heat therapy, monosporic cultures, and nutritional and chemical stress, have been made; however, these methods have not always been successful [44,51,52]. In the case of mitoviruses, finding a cure for the host may be more difficult due to the permanence of the mitochondria.

The replication of mycoviruses as other RNA viruses occurs within a specific optimal temperature range, generally coinciding with the optimal range for host growth. However, mycoviruses replication can be affected by both lower and higher temperatures. The spontaneous elimination of mycoviruses has been observed in various fungal species when the cultures are exposed to lower temperatures or at −80 °C [53]. Generally, lower temperatures inhibit viral replication, as various viral proteins and nucleic acids are synthesized at a specific temperature range [54]. Furthermore, the exposure of fungal isolates to higher temperatures can also impair virus persistence and replication by interfering with critical biological and cellular mechanisms. Moreover, higher temperatures are thought to exacerbate heat shocks and induce protein misfolding within the cell [55], potentially leading to viral loss due to the overexertion of Hsp90, a protein essential for viral replication [56]. However, the exposure of isolates to higher temperatures effectively disrupts viral mobility and interferes with virus-encoded proteins [52,57].

In our study, the effect of thermal treatment on the mitovirus FcMV1differed among isolates. All three isolates of *F. circinatum* (Fc179, 001, and Va221) exhibited a reduction in viral accumulation following thermal treatment, as confirmed through fold change values (0.56, 0.24, and 0.39) corresponding to reductions of 56%, 24%, and 39%, respectively. The observation that different isolates showed different levels of viral reduction (56%, 24%, and 39%) suggests that host genetic factors also influence how temperature affects viral replication. This aligns with previous findings on the relationship between temperature and viral concentration. It has been shown that CHV1 accumulation tends to be higher within a temperature range of 15 to 25 °C, while lower or higher temperature ranges tend to suppress viral accumulation [58]. Furthermore, the efficacy of thermal exposure in eliminating mycoviruses mostly depends on the fungal species. For instance, in the saprotrophic fungus *Heterobasidion ecrustosum*, thermal exposure at 33 °C successfully cured the virus, whereas the fungus was still able to grow at this temperature but at a considerably reduced rate [59,60]. However, similar attempts have failed in other mycoviruses such as *Alternaria alternata* partitivirus 1 (AtPV1), *Sclerotinia sclerotiorum* mitovirus 1 (SsMV1/KL-1), and SsMV2/KL-1 [61]. In contrast, in *Malassezia sympodialis*, which harbors Malassezia sympodialis mycovirus 1 (MsMV1), exposure to high temperatures (37 °C) successfully cured the virus due to the inhibition of various biological functions at higher temperatures [55]. Additionally, the relationship between temperature and viral replication is crucial to understand, as it directly impacts the potential use of mitoviruses as biological control agents in different environmental conditions.

The antiviral ribavirin usually induces mutations in RNA viral genomes and is widely used to combat human viruses such as SARS-CoV-2 [62]. Its antiviral activity has been found to restrain the access of inosine monophosphate dehydrogenase (IMPDH) to its endogenous substrate (inosine-5-monophosphate) through enzyme inhibition [63]. In our study, the treatment with ribavirin at 24 mg/L effectively reduced viral accumulation in *F. circinatum* isolates, with fold change values of 0.70 for Fc179, 0.51 for 001, and 0.49 for Va221, indicating a notable reduction in viral accumulation. Furthermore, a similar study, using ribavirin at a comparable concentration (20–24 mg/L), effectively cured three viral strains from *Tolypocladium cylindrosporium* [64]. However, these mycoviruses belong to families other than Narnaviridae (namely Totiviridae and Chrysoviridae). The same concentration of ribavirin (24 mg/L) was sufficient to successfully eliminate the infection of *Trichoderma harzianum* mycovirus 1 (ThMV1), an unclassified mycovirus infecting *T. harzianum* [65]. Additionally, Herrero et al. (2013) documented that treatment with ribavirin cured *Lentinula edodes* mycovirus HKB (LeV-HKB) infection in Ledodes, which presented as a co-infection with *Lentinula edodes* partitivirus 1 (LePV1) that persisted after the treatment [64]. Both viruses were eventually lost during the fragmentation of mycelium [66]. Since the concentration utilized in the current investigation reflects the maximal concentration at which ribavirin is physiologically active [67], it may be anticipated that antiviral ribavirin does not inhibit mitochondrial viruses, as in *Aspergillus* species.

The replication of viruses can be disrupted using various chemical treatments, thereby limiting their spread [39]. While various antibiotics or antiviral drugs have been documented for mycovirus infection elimination, no specific chemical agent has been identified to successfully limit the infection. Cycloheximide has been extensively used to eliminate mycoviruses from their hosts, as it affects RNA replication through alterations in the ribosomes of the virus genome, thus inhibiting the translation of the genome [68]. The isolates Fc179, Va221, and 001, when treated with cycloheximide, reduced viral accumulation, as indicated by their respective RQ values of 0.89, 0.65, and 0.46. The partial success of cycloheximide has already been reported in *Pseudogymnoascus destructans* [44]. However, its effect usually depends on the nature of the harboring viruses. According to Elias and Cotty (1996), cycloheximide effectively eliminated *Aspergillus fumigatus* chrysovirus (AfuCV) in *A. fumigatus* [69]. In addition, cycloheximide seems to be more effective against ssRNA than dsRNA viruses, due to the effect of antibiotics-mediated interference by viral components. However, it is unclear whether the viral components are based on the biological nature of the cells or the structure of the genome and proteins [70]. The successful elimination of two virus-like RNA segments using cycloheximide (25 mg/L) and ribavirin (73 mg/L) from the fungus *Pseudogymnoascus destructans* confirmed the efficacy of cycloheximide treatments [71]. In addition, cycloheximide is not always successful in curing all fungal strains, even at higher concentrations [41,72,73].

Only a few studies have examined the potential of kanamycin as an antiviral agent, despite its ability to inhibit protein synthesis by binding to the ribosomal 30S subunit [74]. Kanamycin at 100 mg/L has been shown to successfully eliminate mycoviruses such as *Sclerotinia sclerotiorum* hypovirus 2 (SsHV2) and *Botrytis virus* F (BVF). Still, it failed to eliminate *Fusarium poae* Virus 1 infection [14]. Similarly, kanamycin at a lower concentration (50 mg/L) was ineffective in curing the mycovirus *Ceratobasidium* endornaviruses C (CbEVC), whereas this virus was successfully cured using cycloheximide [36]. In the present study, all the *F. circinatum* isolates treated with kanamycin at 50 mg/L depicted a reduction in viral accumulation, with RQ values of 0.62 for Fc179, 0.63 for 001, and 0.62 for Va221, respectively. These results indicate that kanamycin was effective in reducing FcMV1 mitovirus in isolates of *F. circinatum*. However, a greater reduction in viral accumulation can be achieved by using kanamycin at higher concentrations.

Rifampicin functions as an inhibitor of RNA viruses through the prevention of RNA polymerase binding to DNA [75]. In the present study, treatment with rifampicin and cAMP reduced mycovirus levels in *F. circinatum* isolates, with RQ values of Fc179 (0.82), 001 (0.75), and Va221 (0.48), respectively. However, the curing mechanism of rifampin with cAMP needs to be clarified. Still, a minimal medium supplemented with these chemical agents was found to eliminate oyster mushroom spherical virus (OMSV) and oyster mushroom isometric virus (OMIV) from the edible mushroom culture of *Pleurotus ostreatus* [76]. Overall, both thermals and antibiotic treatments effectively reduced viral accumulation from all isolates of *F. circinatum* in general. Although our efforts to eliminate FcMV1 from *F. circinatum* isolates have yet to yield complete success rates, these findings represent a starting point for optimizing mitovirus elimination strategies in this species and in *Mitovirus* spp. infections in general. The mitoviruses reported thus far can exclusively infect the mitochondria of filamentous fungi, and, in certain cases, they are associated with hypovirulence [8]. However, some mitoviruses do not alter mitochondrial morphology and are only involved in mitochondrial morphological alteration (i.e., fibrous mitochondria), which may be associated with induced hypovirulence [27]. Therefore, mitovirus treatment can aid in modifying mitochondrial DNA and viral genome replication, which can be measured using qPCR while taking the viral load into consideration. Additionally, when curing methods are unsuccessful, isogenic isolates with varying viral titers may serve as an alternative to virus-free isolates. Notably, fungal pathogens with higher viral titers have been observed to exhibit phenotypic differences in colony growth, conidiation capacity, and virulence, compared to isolates with lower viral titers [77,78]. Given that low viral titers may persist even when a mycovirus is presumably cured from a fungal isolate [33,53], reducing viral titers could be the most effective approach for mycovirus studies. Therefore, further studies using the isolates generated in this study would help to characterize fungus–virus interactions and provide insights into virocontrol possibilities for managing PPC disease.

## Figures and Tables

**Table 1:** Mean cycle threshold (Ct) values of two replicates obtained using qPCR for different samples before virus-curing treatment.

Isolates	Mean Ct Values
Fc179	17.7
001	18.2
Va221	19.6

**Table 2:** Single-factor ANOVA representing the interaction between isolates after thermal treatment.

Single-Factor ANOVA						
SUMMARY						
*Groups*	*Count*	*Sum*	*Average*	*Variance*		
Fc179.T	3	89.031	29.677	2.375652		
001.T	3	79.994	26.66467	2.57666		
Va221.T	3	87.631	29.21033	2.928101		
ANOVA						
*Source of Variation*	*SS*	*df*	*MS*	*F*	*p-value*	*F crit*
Between-Groups	15.77235	2	7.886174	3.002193	0.124863	5.143253
Within-Groups	15.76083	6	2.626805			
Total	31.53318	8				

## Data Availability

The original contributions presented in the study are included in the article/Supplementary Materials, further inquiries can be directed to the corresponding authors.

## Supplementary Materials

The following supporting information can be downloaded at: https://www.mdpi.com/article/10.3390/jof11040267/s1. Table S1: FcMV1-specific primers used for qPCR; Table S2: Quantitative analysis of all replicates of samples through qPCR; Figure S1: Melting curve plot showing qPCR amplicon length with intercalating dye qPCR assays confirm the presence of mitovirus (FcMV1). The peaks exhibit melting curves of isolates corresponding to positive samples, quantifying virus presence.

## Abbreviations

Common abbreviations that are used in the text.

Abbreviations	
qPCR	Quantitative polymerase chain reaction
RQ	Relative quantification
CT	Cycle threshold
W1	Week 1
W5	Week 5
PDA	Potato dextrose agar
uL	Microliter
ug	Microgram
min	Minute
mg	Milligram
L	Liters
