# Supplementary material for: Effects of Thermal and Antibiotic Treatments on the Viral Accumulation of FcMV1 in Fusarium circinatum Isolates
Source: J Fungi (Basel). 2025 Mar 31;11(4):267. doi: 10.3390/jof11040267 (PMC12027980; doi:10.3390/jof11040267)
Supplement: Supplementary file 1 [file jof-11-00267-s001.zip › jof-3488894-supplementary.pdf]

Supplementary Materials
Table S1: FcMV1-specific primers used for qPCR (Muñoz-Adalia et al. 2018).

Primer name	Primer sequence (5' - 3')	Amplicon size (bp)
FM*V1BL*-F	AGG TCA ACC TAT GGG AGC AT	282
FM*V1BL*-R	AGA CCA CTT ATT TCT TTC CCTGA	282

Table S2: Quantitative analysis of all replicates of samples through qPCR. Ct *β-tubulin* represents the nucleic acid concentration values for endogenous control, while Ct *V1BL* represents the nucleic acid concentration for virus accumulation at week 1 (W1) and week 5 (Week 5). 'T' represents thermal treatment (38 ºC); 'Rib' represents antibiotic Ribavirin; 'Cyc' represents antibiotic cycloheximide; 'Ka' represents antibiotic kanamycin; and 'R + C' represents antibiotic rifampicin+cAMP.s

**Isolates**	**Ct *V1***	**Ct *β-tubulin***
001.1.T.1W	23,201	21,271
001.2.T.1W	22,307	17,788
001.3.T.1W	25,033	19,142
001.1.T.5W	25,415	20,271
001.2.T.5W	26,104	21,788
001.3.T.5W	28,475	20,142
Fc179.1.T.1W	25,071	22,404
Fc179.2.T.1W	21,919	19,731
Fc179.3.T.1W	21,287	17,889
Fc179.1.T.5W	31,083	26,277
Fc179.2.T.5W	29,919	24,731
Fc179.3.T.5W	28,029	23,392
Va221.1.T.1W	23,663	19,439
Va221.2.T.1W	22,722	18,997
Va221.3.T.1W	23,108	17,31
Va221.1.T.5W	27,881	22,95
Va221.2.T.5W	28,609	22,92
Va221.3.T.5W	31,141	29,507
001.1.Rib.1W	19,869	20,743
001.2.Rib.1W	22,822	18,95
001.3.Rib.1W	20,548	19,473
001.1.Rib.5W	26,745	22,917
001.2.Rib.5W	29,81	21,887
001.3.Rib.5W	30,473	22,548
001.1.Cyc.1W	23,382	19,477
001.2.Cyc.1W	23,561	20,228
001.3.Cyc.1W	23,319	22,106
001.1.Cyc.5W	28,893	24,959
001.2.Cyc.5W	27,01	22,738
001.3.Cyc.5W	28,183	23,235

001.1.Ka.1W	22,621456	19,622696
001.2.Ka.1W	22,24564	18,92113
001.3.Ka.1W	24,75632	20,516014
001.1.Ka.5W	27,621456	22,628017
001.2.Ka.5W	28,24564	23,565851
001.3.Ka.5W	26,75632	25,184124
001.1.R+C.1W	20,076889	18,078098
001.2.R+C.1W	23,101185	17,691776
001.3.R+C.1W	20,16571	19,158318
001.1.R+C.5W	26,034908	22,99593
001.2.R+C.5W	27,3972225	24,850637
001.3.R+C.5W	28,072393	21,716341
Fc179.1.Rib.1W	24,225285	20,95834
Fc179.2.Rib.1W	23,02863	18,825943
Fc179.3.Rib.1W	20,392998	17,70264
Fc179.1.RIb.5W	29,62872	24,421852
Fc179.2.RIb.5W	27,10001	25,421852
Fc179.3.RIb.5W	26,99482	23,029
Fc179.1.Cyc.1W	26,6498	24,017902
Fc179.2.Cyc.1W	26,428	23,125
Fc179.3.Cyc.1W	25,525848	23,927227
Fc179.1.Cyc.5W	28,747963	25,1892
Fc179.2.Cyc.5W	27,942638	22,097812
Fc179.3.Cyc.5W	26,931274	23,75623
Fc179.1.Ka.1W	22,172245	22,924349
Fc179.2.Ka.1W	24,210556	19,955639
Fc179.3.Ka.1W	23,739426	21,907646
Fc179.1.Ka.5W	26,210556	19,955639
Fc179.2.Ka.5W	28,210556	23,237007
Fc179.3.Ka.5W	27,74562	22,29499
Fc179.1.R+C.1W	19,355167	18,8979
Fc179.2.R+C.1W	21,158318	22,66378
Fc179.3.R+C.1W	22,146114	19,190512
Fc179.1.R+C.5W	28,15672	22,17218
Fc179.2.R+C.5W	27,43241	19,218
Fc179.3.R+C.5W	26,716341	23,218
Va221.1.Rib.1W	21,163773	21,059227
Va221.2.Rib.1W	24,683304	18,905127
Va221.3.Rib.1W	23,72836	17,064766
Va221.1.Rib.5W	26,152824	23,691776
Va221.2.Rib.5W	27,097113	22,421852
Va221.3.Rib.5W	28,827824	24,468025
Va221.1.Cyc.1W	19,983936	20,204453
Va221.2.Cyc.1W	21,899546	18,480886
Va221.3.Cyc.1W	24,397318	21,069157

Va221.1.Cyc.5W	30,737955	24,474894
Va221.2.Cyc.5W	27,793669	22,290878
Va221.3.Cyc.5W	28,200676	22,005857
Va221.1.ka.1W	20,99524	19,33443
Va221.2.ka.1W	21,997858	19,220528
Va221.3.ka.1W	23,668663	18,418945
Va221.1.ka.5W	26,667103	22,34636
Va221.2.ka.5W	28,667103	23,34636
Va221.3.ka.5W	27,096855	24,460453
Va221.1.R+C.1W	23,01672	19,527155
Va221.2.R+C.1W	21,679363	19,002605
Va221.3.R+C.1W	22,901314	21,436785
Va221.1.R+C.5W	28,59309	20,492355
Va221.2.R+C.5W	29,705032	23,23838
Va221.3.R+C.5W	27,078098	25,100876
FcCa6-positive	24,24762	21,25703
FcCa6-positive	24,25112	23,02612

Figure S1: Melting curve plot showing qPCR amplicon length with intercalating dye qPCR assays confirm the presence of mitovirus (FcMV1). The peaks exhibit melting curves of isolates corresponding to positive samples, quantifying virus presence.

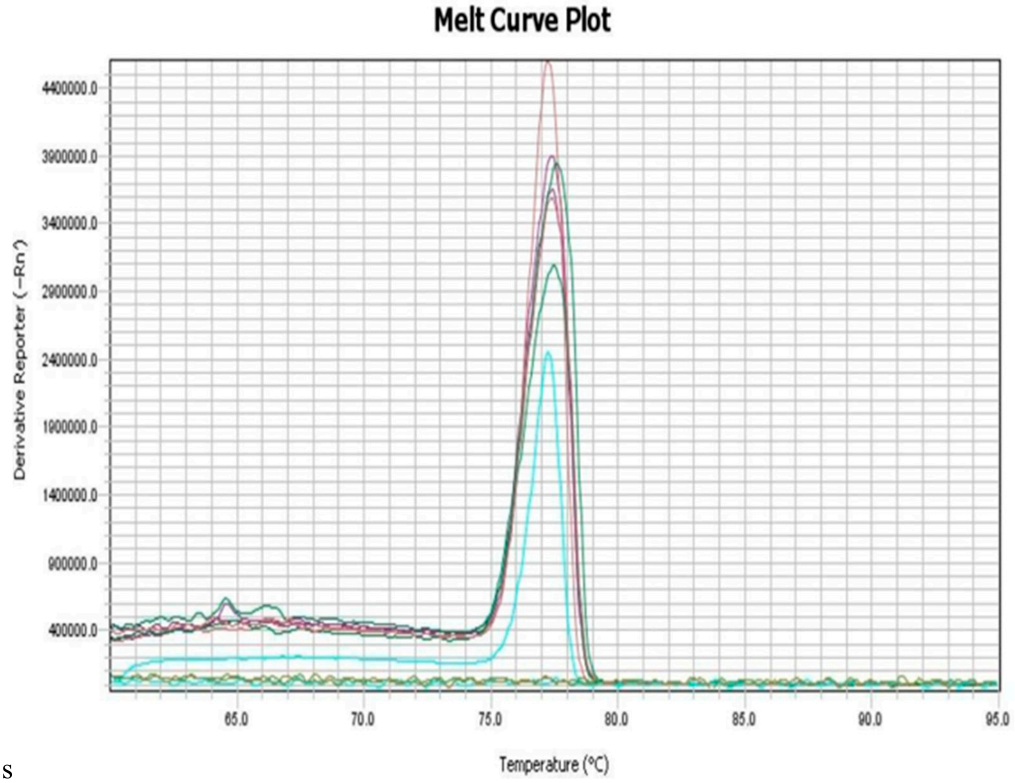

s
